# Cost-Effectiveness of the Pharmacist-Managed Warfarin Therapy vs. Standard Care for Patients With Mechanical Mitral Valve Prostheses: An Egyptian Healthcare Perspective

**DOI:** 10.3389/fcvm.2022.889197

**Published:** 2022-07-13

**Authors:** Radwa Ahmed Batran, Nagwa Ali Sabri, Ihab Ali, Sarah Farid Fahmy

**Affiliations:** ^1^Department of Clinical Pharmacy, Faculty of Pharmacy, Ain Shams University, Cairo, Egypt; ^2^Department of Cardiothoracic Surgery, Cardiothoracic Surgery Academy, Ain Shams University, Cairo, Egypt

**Keywords:** cost-effectiveness, pharmacist intervention, mechanical mitral valve, warfarin, time in therapeutic range, Egypt

## Abstract

**Background:**

Despite warfarin therapy had been used for decades for patients with mechanical mitral valve prostheses (MMVPs), serious and life-threatening complications are still reported worldwide with a significant economic burden. This study is aimed at assessing the clinical and the cost-effectiveness of adopting pharmacist-managed warfarin therapy (PMWT) services for optimizing warfarin treatment in Egypt.

**Methods:**

A prospective randomized trial in which 59 patients with MMVPs were randomly assigned to receive the PMWT services or the standard care and followed up for 1 year. The primary outcome was percentage time in the therapeutic range (TTR). For the cost-effectiveness analysis, a Markov cohort process model with nine mutually exclusive health states was developed from a medical provider’s perspective. A lifetime horizon was applied. All costs and outcomes were discounted at 3.5% annually.

**Results:**

The study results revealed a significantly higher median TTR in the intervention group as compared to the control group; 96.8% [interquartile range (IQR) 77.9–100%] vs. 73.1% (52.7–95.1%), respectively, *p* = 0.008. A significant association between standard care and poor anticoagulation control (*p* = 0.021) was demonstrated by the multivariate regression analysis. For the cost-effectiveness analysis, the total cumulative quality-adjusted life-years (QALYs) and total costs per patient were 21.53 and 10.43; 436.38 and 1,242.25 United States dollar (USD) in the intervention and the control groups, respectively, with an incremental cost-effectiveness ratio (ICER) of −72.5796 for the intervention group.

**Conclusion:**

The PMWT strategy was proven to provide a significantly better anticoagulation control and to be a cost-saving approach in Egyptian patients with MMVPs. Nevertheless, the dominance of this strategy is sustained by maintaining the therapeutic International Normalized Ratio (INR) control within the recommended range. Our findings will benefit Egyptian policy-makers who may seek novel health strategies for better resource allocation.

**Clinical Trial Registration:**

[ClinicalTrials.gov], identifier [NCT04409613].

## Introduction

Rheumatic heart disease (RHD) is the most prevalent heart condition in children and adolescents who are aged 25 or below. So far, RHD deems endemic in many low- and middle-income countries among vulnerable groups (1, 2). The highest RHD deaths were reported from Egypt, Yemen, Pakistan, Afghanistan, and Iran, which count for 80% of the total death rates for the Eastern Mediterranean Region (3). Almost 20% of patients with RHD become afflicted by congestive heart failure that requires valve surgery within 5–10 years (4). The surgical replacement of a heart valve with a mechanical prosthetic one aims to restore heart function; however, the implantation of a mechanical valve is an absolute indication for lifelong oral anticoagulation therapy to avert the risk of potential complications, such as bleeding, thromboembolic events, prosthetic valve endocarditis, and dysfunction. Moreover, the risk of extensive anticoagulation, i.e., to effectively avoid thromboembolic events, has to be weighed against another risk of bleeding complications (5, 6).

Vitamin K antagonists (VKAs) are the unique recommended option for oral anticoagulation in patients with mechanical valves (7). Among VKAs, warfarin is the most frequently prescribed drug that had been used for decades for those patients. However, it is still challenging to maintain a safe and efficient treatment (8). Major bleeding (which can be life-threatening), intracranial bleeding, and fatal bleeding are observed in 2–5, 0.2–0.4, and 0.5–1.0%, respectively, with patients on warfarin per year (9). What is more, warfarin therapy is fraught with several inherent problems, such as great diversity in dosing, delayed onset of action along with prolonged effect after discontinuation, a wide range of serious interactions, and a narrow therapeutic index. Collectively, all these problems designate warfarin as a “high alert medication” that calls for extraordinary caution and care (10–14).

There is growing evidence that the management of anticoagulation by experienced pharmacists can lead to better outcomes as compared to standard care. This is backed by data from multiple studies that reported improved clinical outcomes with the pharmacist-managed warfarin therapy (PMWT) services, such as significantly better International Normalized Ratio (INR) control, and significant reductions in rates of emergency department visits, hospitalizations, hospital length of stay, and hospital readmission rates. In consequence, cost savings have been well demonstrated in studies from different sides of the world due to decreased rates of both adverse events and complications, with subsequent impact on medical and non-medical costs (12, 15–21). The objective of this trial-based economic evaluation study was to evaluate the clinical effectiveness and the cost-effectiveness of the PMWT services for outpatients with mechanical mitral valve prostheses (MMVPs) in an Egyptian University Teaching Hospital setting.

## Patients and Methods

### Study Design and Participants

This was a prospective randomized controlled study in which patients with MMVPs were recruited from the outpatients’ anticoagulation clinic at the Cardiothoracic Surgery Academy, Ain Shams University, Cairo, Egypt and were randomly assigned to either the control group, who received standard medical care or the intervention group, who received the PMWT services. Patients were considered for eligibility in the study if they had met the following inclusion criteria: (1) men or women between 18 and 70 years of age, (2) post-mitral valve surgery patients with MMVPs, and (3) patients with a prescription of warfarin. Although pregnant patients, patients with double or aortic valve replacement surgery, patients with biological prostheses, and patients with congenital blood disorders were excluded from the study.

### Methods

Starting 1 February 2020, a total of 107 patients were screened for eligibility in the study, of which, 48 patients were excluded, as they did not meet the inclusion criteria. Thus, a total of 59 patients were included and randomly allocated to either the intervention (patients who received the PMWT, *n* = 29) or the control (patients who received the standard medical care, *n* = 30) groups [a Consolidated Standards of Reporting Trial (CONSORT) flow diagram is provided in the Supplementary Figure 1]. During the first patient visit, demographic data were collected which included name, age, gender, weight, height, education, occupation, residence, and lifestyle habits (smoking and level of physical activity). Along with these data, previous and current medications, comorbidities, and INR test results were also collected. Eligible patients in both groups were followed up for 1 year.

#### Standard Medical Care

Standard medical care included the documentation of INR test results, and providing the patients with instructions regarding warfarin dose, regimen, any dose modification, and frequency of INR testing. All these services were provided by the anticoagulation clinic staff (which consisted of both cardiologists and nurses).

#### Pharmacist-Managed Warfarin Therapy Services

The PMWT services included two main pillars, which are patients’ education and counseling and patients’ follow-up. Patients in the intervention group (or their caregivers) received an educational session, which was aimed to ensure that the patient understands the risks, the required precautions, and the necessity for frequent monitoring. Education involves assessing the patient’s understanding of his health problems, the ability to take warfarin correctly, and attitudes toward any warfarin-related complications.

Multiple methods were applied during this session, such as asking open-ended questions to evaluate the patient’s understanding and subsequently, to decide what information is needed to be provided. Visual aids were also used to promote the patient’s comprehension. Therefore, the information was presented as an educational leaflet (Supplementary Figure 2) and explained to the patient in simple language. The information provided in the leaflet included the drug’s brand and generic name, purpose, anticipated onset, frequency of dosing, important precautions, lifestyle modifications, common side effects and what to do if they occur, the importance of regular monitoring and proper adherence, and the need to inform provider if the patient is planning to become pregnant, before any procedure or hospitalization and before starting any new drug. At subsequent visits, this information was briefly recalled to refresh the patient’s information.

Patients were followed up from February 2020 to February 2021. They were regularly assessed through INR value recording, warfarin dose, and any warfarin-related problems, with further dose readjustment recommendations according to the patient’s INR test result. Signs of warfarin overdose were also reported. The time interval for measuring INR levels was typically 1 month. However, this was not constant on a practical basis and the time interval between successive patient visits was primarily based on INR test results.

### Primary End Point

The primary end point was the percentage time in therapeutic range (TTR), i.e., the proportion of time spent within the target INR range (22), and was calculated by the Rosendaal method (23). TTR below 65% was considered to be associated with poor anticoagulation control as per the 2020 National Institute for Health and Care Excellence (NICE) recommendations (24). An INR range of 2.5–3.5 was recommended as per the 2020 American College of Cardiology/American Heart Association Guideline for the Management of Patients With Valvular Heart Disease (7).

### Ethical Approval

This study was performed following the principles of the Declaration of Helsinki (25). Ethical approval was granted by the Research Ethics Committee at the Faculty of Pharmacy, Ain Shams University, Cairo, Egypt (ENREC–ASU, 2019-99). Informed consent was obtained from each participant without any obligation to complete the study if they did not want to.

### Sample Size Calculation and Statistical Methods

Reference to Falamić et al. (8), in which the median TTR was significantly higher in the group that received PMWT services than the standard care (93% vs. 31.2%, a difference of 61.8%), a total of 36 patients (18 per group) were required. The sample size was calculated using Stata/ES 14.2 software for windows. IBM SPSS program (version 20) was used to perform the statistical analysis. All graphs were done using Microsoft Excel 2016. All *p*-values were two-sided, and the difference was considered statistically significant if the *p*-value was ≤ 0.05 [95% confidence intervals (CI); detailed sample size calculation and statistical methods are provided in Supplementary Figure 3].

### Economic Evaluation

#### Overview

**FIGURE 1:**
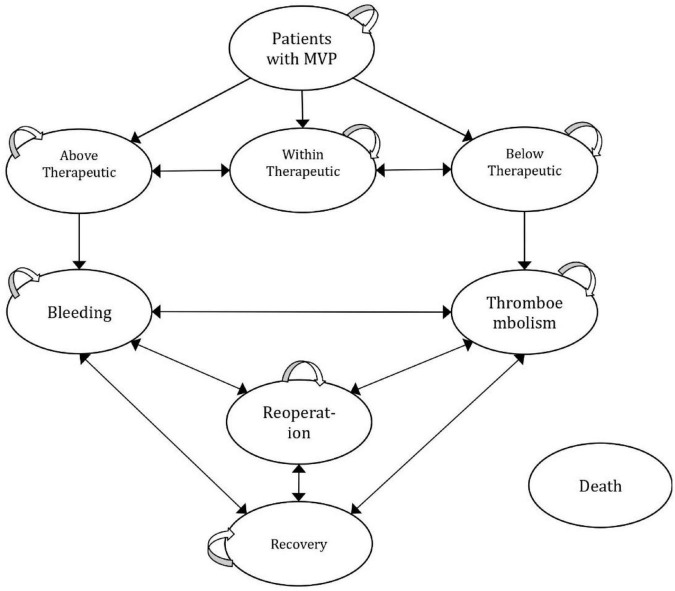
Schematic diagram for the Markov state transition.

Figure 1 represents a half-cycle-corrected Markov cohort process model, which is developed to reflect the real practice of patient management in Egypt and risks that change repeatedly over time, with nine mutually exclusive health states (25). The model structure and inputs were verified by clinical experts and a lifetime horizon was selected to reflect the long-term consequences. A 1-month cycle length was adopted. The model was populated with relevance to a medical provider’s perspective. All costs and outcomes were discounted at 3.5% annually (26). The analysis was performed using Microsoft Excel 2016.

#### Model Inputs and the Likelihood of Events

Patients entered the Markov model by either receiving the PMWT services or the standard care. The health states included in the model were “within therapeutic range,” “below therapeutic range,” and “above therapeutic range,” which were defined as INR values below 2.5, within 2.5–3.5, and above 3.5, respectively (27). The “bleeding” state was defined by a major bleeding event that required hospitalization or blood transfusion; the “thromboembolism (TE)” state was defined by the occurrence of a thromboembolic event with morbidity that included ischemic stroke/transient ischemic attack, deep vein thrombosis, and pulmonary embolism; the “reoperation” state was defined as patients who underwent reoperation; and the “recovery” state was defined as the patients recovered after an event; and death, which was defined as death from any cause (27, 28).

The transition probabilities used in the model are shown in Table 1. To reflect the Egyptian population, the cohort that entered the model was transitioned to within, below, or above therapeutic range states based on probabilities derived from the Cardiothoracic Surgery Academy, Cairo, Egypt. With every cycle, the patient could remain in the current state or transit between below, within, and above the therapeutic range back and forth. Afterward, the patients could stay in the no-event state or experience the following events: bleeding, TE, reoperation, or death from any cause. The model accounts for the risk of mortality at all states of any warfarin-associated events. Death risk ratios from major TE or bleeding events (i.e., additional to death from any cause) were also added to the model.

**TABLE 1:** Model inputs and data sources for the cost-effectiveness analysis.

Parameter	Base case	Range		Source(s)
		
		Low value	High value	
**Initial population proportion in the intervention group (no-event states)**				
Within therapeutic range	0.9942	0.8948	1.0937	CTSA
Below therapeutic range	0.0029	0.0026	0.0032	CTSA
Above therapeutic range	0.0029	0.0026	0.0032	CTSA
**Initial population proportion in the control group (no-event states)**				
Within therapeutic range	0.9855	0.8869	1.0840	CTSA
Below therapeutic range	0.0058	0.0052	0.0063	CTSA
Above therapeutic range	0.0087	0.0079	0.0096	CTSA
**INR transition probabilities in the intervention group**				
Within therapeutic to below therapeutic	0.0225	0.0203	0.0248	CTSA
Within therapeutic to above therapeutic	0.0191	0.0172	0.0210	CTSA
Below therapeutic to within therapeutic	0.4263	0.3837	0.4689	CTSA
Above therapeutic to within therapeutic	0.5712	0.5141	0.6283	CTSA
**INR transition probabilities in the control group**				
Within therapeutic to below therapeutic	0.0741	0.0667	0.0815	CTSA
Within therapeutic to above therapeutic	0.0383	0.0345	0.0421	CTSA
Below therapeutic to within therapeutic	0.1589	0.1430	0.1748	CTSA
Above therapeutic to within therapeutic	0.291	0.2619	0.3201	CTSA
Above therapeutic to bleeding	0.0115	0.0104	0.0127	(29)
Below therapeutic to TE	0.0023	0.0021	0.0025	(29)
TE to bleeding	0.00228	0.00205	0.00251	(28)
TE to reoperation	0.00033	0.00030	0.00037	(28)
TE to death	0.00374	0.00337	0.00412	(28)
TE to TE	0.08825	0.07942	0.09707	(28)
Bleeding to TE	0.00059	0.00053	0.00064	(28)
Bleeding to reoperation	0.00033	0.00030	0.00037	(28)
Bleeding to death	0.01078	0.00971	0.01186	(28)
Bleeding to bleeding	0.02049	0.01844	0.02254	(28)
Reoperation to TE	0.00014	0.00012	0.00015	(32)
Reoperation to bleeding	0.00028	0.00025	0.00031	(32)
Reoperation to death	0.00035	0.00031	0.00038	(32)
Reoperation to reoperation	0.00033	0.00030	0.00037	(28)
Recovery to TE	0.00059	0.00025	0.00109	(33)
Recovery to bleeding	0.00228	0.00109	0.00532	(33)
Recovery to reoperation	0.00033	0.00017	0.00075	(30, 31)
Recovery to death	0.00147	0.00131	0.00163	(34)
**Death risk ratios (additional to death with no-events)**				
Major TE	2.25	1.75	2.75	(27, 29)
Major bleeding	1.5	1	2	(27, 29)
**Utilities**				
Within therapeutic	0.987	0.967	0.998	(27)
Bleeding	0.54	0.44	0.74	(36)
TE	0.45	0.35	0.55	(29)
Reoperation	0.45	0.35	0.75	(36)
Recovery	0.668	0.61	0.76	(29)
**Costs**				
Cost of bleeding event (USD)	2,777.78	2,500.00	3,055.56	CTSA
Cost of TE event (USD)	2,314.81	2,083.33	2,546.30	CTSA
Cost of reoperation event (USD)	16,203.70	14,583.33	17,824.07	CTSA
Discount rate of cost	0.035	0.02	0.06	(26)
Discount rate of QALY	0.035	0.02	0.06	(26)

*Transition probabilities are shown as probabilities per 1-month cycle.*

*CTSA, Cardiothoracic Surgery Academy, Ain Shams University, Cairo, Egypt; INR, international normalized ratio; QALY, quality-adjusted life year; TE, thromboembolism; USD, United States dollar.*

Due to the lack of local data, we used the probabilities of developing major TE and bleeding events, and death risk ratios after major TE or bleeding events, using published pooled data from several sources (29). The risk for reoperation was obtained from two clinical trials that included 394 and 211 patients with mitral valve replacement (MVR) and patients who were randomized to receive either biological or mechanical valves (30, 31). The probabilities of bleeding, TE, or death with reoperation were derived from a published review that compared the results of 106 patients who underwent repeat MVR with 562 control patients who underwent primary replacement using a computerized database. These risks were calculated using a multivariate logistic regression model to predict associated events or mortality (32). The risks of both major bleeding and TE events after recovery were derived from a large study that included a total of 1,608 patients who were followed for 6,475 patient-years to determine the incidence of complications of oral anticoagulation therapy in patients with mechanical valves (33). The risk of mortality after a recovery state was developed from a retrospective review of 671 patients’ hospital records over 9 years (34). The transition probabilities from the recovery state to reoperation, bleeding, and TE were derived from a previous decision analysis that was modeled on Egyptian patients and assumed that these probabilities are the same as those for patients in the reoperation state (28).

#### Outcomes

The outcomes were measured in terms of quality-adjusted life-years (QALYs) for both groups. This generic measurement is commonly used as a summary measure for economic evaluations, which combines the impact on both the quality and quantity of life into a single parameter (35). The utilities within the therapeutic range, bleeding, reoperation, recovery, and TE states incorporated in the model were obtained from different published studies (27, 29, 36).

#### Costs

From a medical provider’s perspective, only direct medical care costs were taken into account, i.e., the costs of bleeding, reoperation, and TE events. All cost inputs were obtained from the databases of the Cardiothoracic Surgery Academy, Ain Shams University, Cairo, Egypt. A macro-costing approach was applied. The costs of INR testing and warfarin were not included since these costs are not covered by the medical provider. We assumed no difference in resource use between the two strategies. Conversion of the local Egyptian currency to United States dollar (USD) was performed using the purchasing power parity rate. All costs were calculated in USD for the financial year of 2019 (37).

#### Sensitivity Analysis

To test the robustness of our model results across variations in input estimates, one-way sensitivity analyses were performed as recommended by the Consolidated Health Economic Evaluation Reporting Standards (CHEERS): The International Society for Pharmacoeconomics and Outcomes Research (ISPOR) Task Force report (38). The stability of the model was tested for all estimates of clinical parameters, utilities of health states, costs, and discount rates. All inputs were varied across upper and lower limits for each parameter. These parameters were delineated by CIs from the literature or reasonable ranges that were determined based on different published sources. Microsoft Excel 2016 was used to perform all analyses.

## Results

### Study Population

A total of 59 patients were included in the study, of which, 29 patients (49.2%) were included in the intervention group and 30 patients (50.8%) in the control group. Detailed demographic and clinical characteristics of the study participants are illustrated in Table 2. There was no statistically significant difference between both groups in terms of baseline characteristics.

**TABLE 2:** Baseline demographic data of the study participants.

Parameter	Total (*n* = 59)	Intervention group (*n* = 29)	Control group (*n* = 30)	*P*-value
Age (years)	46.41 ± 10.53	46.79 ± 10.96	46.03 ± 10.28	0.785
**Gender**				
Males	17 (28.8%)	8 (27.6%)	9 (30%)	0.838
Females	42 (71.2)	21 (72.4%)	21 (70%)	
Weight (kg)	75 (62–85)	75 (60–85)	73 (64.5–85.25)	0.632
Height (cm)	162 (159–170)	164 (155–170)	161 (159.8–166.3)	0.819
BMI (kg/m^2^)	27.84 ± 5.02	27.27 ± 4.80	28.38 ± 5.25	0.400
**Residence**				
Urban	26 (44.1%)	15 (51.7%)	11 (36.7%)	0.244
Rural	33 (55.9%)	14 (48.3)	19 (63.3%)	
**Education**				
Illiterate	26 (44.1%)	12 (41.4%)	14 (46.7%)	0.621
Primary	12 (20.3%)	5 (17.2%)	7 (23.3%)	
Secondary	14 (23.7%)	9 (31%)	5 (16.7%)	
University	7 (11.9%)	3 (10.3%)	4 (13.3%)	
**Occupation**				
Employed	17 (28.8%)	9 (31%)	8 (26.7%)	0.711
Unemployed	42 (71.2%)	20 (69%)	22 (73.3%)	
**Smoking**				
Never	47 (79.7%)	24 (82.8%)	23 (76.7%)	0.796
Former	9 (15.3%)	4 (13.8%)	5 (16.7%)	
Current	3 (5.1%)	1 (3.4%)	2 (6.7%)	
**Physical Activity**				
Inactive	3 (5.1%)	2 (6.9%)	1 (3.3%)	0.162
Low	22 (37.3%)	8 (27.6%)	14 (46.7%)	
Moderate	27 (45.8%)	17 (58.6%)	10 (33.3%)	
High	7 (11.9%)	2 (6.9%)	5 (16.7%)	
**Comorbidities**				
Number of comorbidities	1 (1–2)	1 (1–2)	1 (1–2)	—–*^a^*
Heart failure	42 (71.2%)	19 (65.5%)	23 (76.7%)	0.344
Stroke	3 (5.1%)	2 (6.9%)	1 (3.3%)	0.530
Hepatic	0 (0%)	0 (0%)	0 (0%)	—–*^b^*
Gastrointestinal	5 (8.5%)	4 (13.8%)	1 (3.3%)	0.137
Acute kidney injury	4 (6.8%)	3 (10.3%)	1 (3.3%)	0.275
Asthma	1 (1.7%)	1 (3.4%)	0 (0%)	0.230
Diabetes mellitus	5 (8.5%)	3 (10.3%)	2 (6.7%)	0.611
**Obesity*^c^***				
Normal	21 (35.6%)	11 (37.9%)	10 (33.3%)	0.735
Pre-obesity	19 (32.2%)	11 (37.9%)	8 (26.7%)	
Obesity I	14 (23.7%)	5 (17.2%)	9 (30%)	
Obesity II	3 (5.1%)	1 (3.4%)	2 (6.7%)	
Obesity III	2 (3.4%)	1 (3.4%)	1 (3.3%)	

*Results are expressed as mean ± standard deviation (SD), median (interquartile range), or frequency (%).*

*The statistical test used is the Mann–Whitney U test for numerical data or the chi-square test for categorical variables.*

*A p-value ≤ 0.05 is considered statistically significantly different from the control [95% confidence interval (CI)].*

*BMI, body mass index.*

*^a^The distribution is the same across categories. No statistics are computed.*

*^b^No measures of association are computed because the variable is constant.*

*^c^Obesity was categorized according to the body mass index as follows; normal weight 18.5–24.9, pre-obesity 25.0–29.9, obesity class I 30.0–34.9, obesity class II 35.0–39.9, and obesity class III above 40 (39).*

### Time in Therapeutic Range

By the end of the follow-up period, the median TTR was significantly higher in the intervention group than in the control group (*p* = 0.008) indicating that patients who received the PMWT services spent significantly more time in the therapeutic range. For TTR categories, the percentage of patients with poor INR control, i.e., TTR < 65%, was also significantly higher in the control group (*p* = 0.011). On the other hand, PMWT services were associated with a significantly higher percentage of patients with controlled TTR > 75% as compared to the control group (*p* = 0.004). TTR-related outcomes are illustrated in Table 3 (additional figures are illustrated in Supplementary Figures 4, 5). Red spots, bleeding gums, and nose bleeding were decreased in patients who received the PMWT services as compared to the control group (7.1% vs. 13.8%, 3.4% vs. 6.9%, and 6.9% vs. 10.3%, respectively); however, the differences did not reach statistical significance (*p* > 0.05).

**TABLE 3:** TTR outcomes at the end of the follow-up period.

Parameter	Intervention group (*n* = 28)	Control group (*n* = 27)	*P*-value	Significance
TTR (%)	96.8% (77.9–100%)	73.1% (52.7–95.1%)	0.008	**Significant**
TTR categories				
<65%	3 (10.7%)	11 (40.7%)	0.011	**Significant**
65%–75%	3 (10.7%)	5 (18.5%)	0.410	N.S
>75%	22 (78.6%)	11 (40.7%)	0.004	**Significant**

*Results are expressed as median (interquartile range) or frequency (%).*

*The statistical test used: Mann–Whitney U test for numerical data or chi-square test for categorical variables.*

*A p-value ≤ 0.05 is considered statistically significantly different from the control [95% confidence interval (CI)].*

*TTR, time in therapeutic range; N.S, not significant.*

### The Association Between Standard Care and Poor Quality of Anticoagulation (TTR < 65%): A Multivariate Logistic Regression Analysis

The results of the multivariate logistic regression analysis (Table 4) show that receiving the standard care is significantly associated with poor INR control (dependent variable is TTR < 65%), with an odds ratio (OR) of 6.53 [95% CI (1.33–32.19)], and *p* = 0.021. These results were obtained while controlling other covariates, namely, age, body mass index (BMI), number of comorbidities, heart failure, stroke, and diabetes (a forest plot diagram is illustrated in Supplementary Figure 6).

**TABLE 4:** Risk of poor quality of anticoagulation (TTR < 65%) associated with standard care: a multivariate logistic regression analysis.

Predictors	Multivariate Analysis			
	
	*p*-value	OR	95% CI for OR	
			
			Lower	Upper
Standard care (control group)	0.021*	6.53	1.33	32.19
Age	0.797	1.01	0.94	1.08
BMI	0.879	1.01	0.87	1.18
Number of comorbidities	0.524	0.61	0.13	2.83
Heart Failure	0.200	0.18	0.01	2.45
Stroke	0.942	0.90	0.05	17.87
Diabetes	0.956	1.11	0.03	37.14

*The statistical test used: Multivariate logistic regression analysis.*

*p-value ≤ 0.05 considered a statistically significant predictor [95% confidence interval (CI)].

*BMI, body mass index; CI, confidence interval; OR, odds ratio; TTR, time in therapeutic range.*

### Results of the Cost-Effectiveness Analysis

**FIGURE 2:**
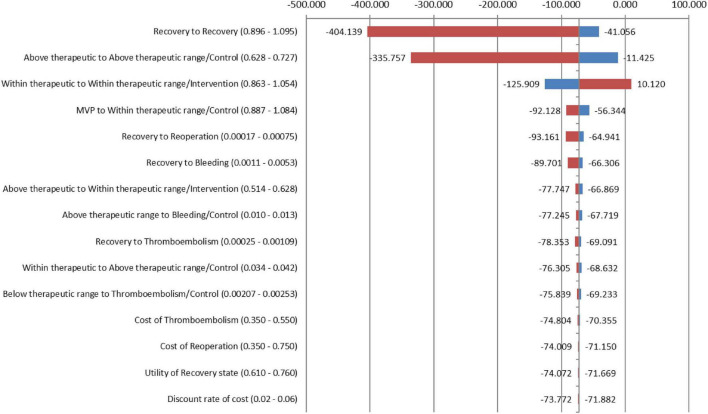
Tornado diagram showing a series of one-way sensitivity analyses.

From the medical provider’s perspective, the total cumulative QALY per patient for the PMWT group was 21.53 when compared with 10.43 for the standard care group. The total cumulative costs per patient for the PMWT and the standard care groups were 436.38 and 1,242.25 USD, respectively. These results yielded an incremental cost-effectiveness ratio (ICER) of − 72.5796 for the intervention group. Thus, the strategy of the PMWT services was dominant, i.e., a cost-saving strategy (Table 5). For the one-way sensitivity analyses, the PMWT strategy would become very cost-effective [based on the threshold stated by the World Health Organization (WHO); 3 × GDP/capita] rather than being cost-saving by a 10% reduction in the probability of remaining within the therapeutic range for the intervention group. When all other inputs were altered between the low and high values, no effects on the resultant ICER values were found. None of the extreme values of any parameter changed the results to be not cost-effective (Figure 2).

**TABLE 5:** Decision analytic model results.

Group	Model results			
	
	Total cumulative costs/patient (USD)	Total cumulative QALYs/patient	ICER	Interpretation
The PMWT (intervention group)	436.38	21.53	**−72.5796**	**Dominant Strategy**
Standard care (control group)	1,242.25	10.43		
Difference	−805.87	11.10		

*ICER, incremental cost-effectiveness ratio; PMWT, pharmacist-managed warfarin therapy; QALY, quality-adjusted life-year; USD, United States dollar.*

## Discussion

This study was a trial-based cost-effectiveness evaluation for adopting the PMWT services to patients with MMVPs on warfarin, which revealed that this strategy is cost-saving in the Egyptian setting. To the best of our knowledge, this is the first study to provide this evidence in Egypt. We believe that this evidence could be used to target further quality improvement efforts, which can ultimately enhance both clinical outcomes and cost-saving attempts in an era when a better allocation of scarce resources is at the precedence of healthcare policy initiatives.

As has been shown, the PMWT services have demonstrated a substantial improvement in patients’ anticoagulation control as compared to standard care. This was confirmed by the significantly higher median TTR recorded with the PMWT services (96.8% in the intervention group vs. 73.1% in the control group, *p* = 0.008), which indicates a significant increase in time spent under the target INR range. Our study results are in accordance with the results of a recent systematic review that was conducted by Entezari-Maleki et al. (40). They studied a total of 4 randomized controlled trials (RCTs) and 20 non-RCT studies with total included patients 11,607 to compare the potential benefit of PMWT as compared to usual medical care. Their results revealed significant improvement in TTR (72.1% vs. 56.7%; *p* = 0.013) in favor of the PMWT. Moreover, significant differences were reported in major bleeding events, thromboembolic events, hospitalization, emergency department visits in favor of the PMWT as compared to usual medical care (0.6% vs. 1.7%, *p* < 0.001; 0.6% vs. 2.9%, *p* < 0.001; 3% vs. 10%, *p* < 0.001; and 7.9% vs. 23.9%; *p* < 0.0001, respectively).

Another study was conducted by Marcatto et al. (11) to evaluate the impact of pharmacist’s warfarin management in 268 patients with poor quality of anticoagulation (TTR < 50%). They applied a different design by comparing the retrospective data for the included patients with data obtained prospectively with the same patients after assigning them to receive the pharmacist’s warfarin management services. The investigators reported a statistically significant increase in TTR after 4 and 12 weeks as compared to basal TTR. Furthermore, the mean TTR 1 year before (retrospective phase) was significantly lower than the TTR reported after 12 weeks of pharmacist-driven treatment (prospective phase; *p* < 0.001). Similarly, Dib et al. (41) conducted a study to evaluate the impact of the first pharmacist-managed anticoagulation clinic in the eastern province of Saudi Arabia. The authors reported that the total percentage of INR within the target range was 59% vs. 48% with the pharmacist-managed clinic vs. the traditional care, respectively. In contrast, Wu et al. (42) evaluated the impact of pharmacist interventions on INR control after MVR during the warfarin initiation phase and demonstrated a numerically higher TTR in the PMWT group as compared to the conventional group, however, the difference did not reach the statistical significance.

In this work, the results of the multivariate logistic regression analysis revealed that standard care was significantly associated with poor quality of anticoagulation; OR 6.53, 95% CI [1.33–32.19], *p* = 0.021. These results were in line with the results reported by Falamic et al. (8). They reported that the pharmacist’s intervention was significantly associated with good quality of anticoagulation control (dependent variable TTR ≥ 65%); OR 77.84, 95% CI [8.25–734.14], *p* < 0.001. Our study results are consistent with the results reported by Wu et al. (42). They revealed that PMWT was associated with achieving therapeutic INR at discharge by the multivariate regression analysis [OR 3.14, 95% CI (1.08–9.14)] and was inversely associated with achieving INRs above the target range during admission [OR 0.21, 95% CI (0.05–0.82)].

In the current study, the results of the cost-effectiveness analysis demonstrated that the PMWT strategy was dominant (i.e., less costly and more effective) as compared to standard care from the medical provider’s perspective. This finding was similar to the results from previous studies conducted in other countries, such as the United States and Thailand, which strongly suggests that this intervention is cost-effective across different types of healthcare systems (15, 27, 43, 44).

In Egypt, RHD has been considered a national healthcare problem since the rate of misdiagnosis of rheumatic fever remains high. Moreover, the mitral valve is the most afflicted site among Egyptian patients (95.2% out of all valvular afflictions). In response, the Egyptian Ministry of Health has established the national RHD prevention and control program in 2006. The program was projected to save 1.7 billion USD, which represents the cost of valve replacement surgeries required for the number of RHD cases if they are neglected (45). In line with these efforts, we believe that our study results advocate for adopting a simple strategy, the PMWT strategy, that ultimately ends up with massive cost savings and better allocation of the scarce healthcare resources in Egypt. Moreover, our study was conducted from the medical provider’s perspective; however, we believe that the adoption of these services will be even more cost-saving from the societal perspective.

This study included several strong points as it incorporated real-life data to demonstrate the clinical effectiveness of the PMWT services as compared to the actual care experienced by patients in day-to-day practice in Egypt. Moreover, this model is the first one in Egypt that incorporated transitions over time through INR changes and translated this in terms of increased risks of developing warfarin-associated events. Additionally, the parameters used in our analysis were derived from large RCTs (30, 31). In the used model, we explicitly accounted for uncertainties of the epidemiologic parameters, relative risks, and quality of life by using ranges and CIs based on published sources.

Our study has several limitations. We did not compare the time to achieve therapeutic INR in both arms. We believe that larger studies are needed to investigate this outcome that is strongly believed to endorse not only the clinical effectiveness of the PMWT but also the cost savings associated with this approach. In our economic model, we assumed that the rate of TE on reoperation is identical to that for patients in the recovery state due to the lack of reliable data. In this context, it is worth mentioning that the incorporation of several simplifying assumptions is considered a weak point for the model; however, this was overcome by the sensitivity analyses that adopted wide ranges for parameter values. In addition, some input parameters were derived from studies that were conducted on different populations due to a lack of local data. However, these parameters did not show any significant impact on the results in the one-way sensitivity analyses. Another additional limitation, we adopted the medical provider’s perspective. Thus, we did not include the loss of productivity costs and the costs incurred by the patient (costs of the drug and the INR testing) as an out-of-pocket cost.

## Conclusion

The pharmacist-managed warfarin therapy strategy was proven to provide a significantly better anticoagulation control and to be a cost-saving approach in Egyptian patients with MMVPs. Nevertheless, the dominance of this strategy is sustained by maintaining the therapeutic INR control within the recommended range. Our findings will benefit Egyptian policy-makers who may seek novel health strategies for better resource allocation.

## Data Availability Statement

The original contributions presented in this study are included in the article/Supplementary Material, further inquiries can be directed to the corresponding author.

## Ethics Statement

The studies involving human participants were reviewed and approved by the Research Ethics Committee at Faculty of Pharmacy, Ain Shams University, Cairo, Egypt (ENREC–ASU. 2019-99). The patients/participants provided their written informed consent to participate in this study.

## Author Contributions

RB and IA performed the practical part at the anticoagulation clinic. RB performed the statistical and economic analysis and wrote the manuscript. SF and NS revised the manuscript. SF, IA, and NS supervised the project. All authors designed the research and read and approved the final manuscript.

## Conflict of Interest

The authors declare that the research was conducted in the absence of any commercial or financial relationships that could be construed as a potential conflict of interest.

## Publisher’s Note

All claims expressed in this article are solely those of the authors and do not necessarily represent those of their affiliated organizations, or those of the publisher, the editors and the reviewers. Any product that may be evaluated in this article, or claim that may be made by its manufacturer, is not guaranteed or endorsed by the publisher.
