# Supplementary material for: Cost-Effectiveness of the Pharmacist-Managed Warfarin Therapy vs. Standard Care for Patients With Mechanical Mitral Valve Prostheses: An Egyptian Healthcare Perspective
Source: Front Cardiovasc Med. 2022 Jul 13;9:889197. doi: 10.3389/fcvm.2022.889197 (PMC9327740; doi:10.3389/fcvm.2022.889197)
Supplement: Supplementary file 1 [file Data_Sheet_1.docx]

**Supplementary Material**

**Cost-Effectiveness of the Pharmacist-Managed Warfarin Therapy versus Standard Care for Patients with Mechanical Mitral Valve Prostheses: An Egyptian Healthcare Perspective**

**Radwa Ahmed Batran^1^, Nagwa Ali Sabri^1*^ , Ihab Ali^2^, and Sarah Farid Fahmy^1^**

^1^Clinical Pharmacy Department, Faculty of Pharmacy, Ain Shams University, African Union Organization Street, Cairo 11566, Egypt.

^2^Cardiothoracic Surgery Department, Cardiothoracic Surgery Academy, Ain Shams University, Ramses Street, Cairo 11355, Egypt.

***Corresponding author:**

Nagwa Ali Sabri

Clinical Pharmacy Department, Faculty of Pharmacy, Ain Shams University, African Union Organization Street, Cairo 11566, Egypt

E-mail address: [nagwa.sabri@pharma.asu.edu.eg](mailto:nagwa.sabri@pharma.asu.edu.eg)

Tel.: +20 122 341 1984

ORCID ID: 0000-0002-2611-4853

**Supplementary Material**

**Fig. S1; CONSORT Flow Diagram**

## Follow-Up

Analysed (n=28)

## Analysis

Analysed (n=27)

Lost to follow-up (due to the COVID19 pandemic) (n=2)

Lost to follow-up (due to the COVID19 pandemic) (n=1)

## Enrollment

Allocated to intervention group (n= 29) and received the PWTM services plus standard care

## Allocation

Allocated to control group (n= 30) and received the standard care alone

Randomized (n= 59)

Excluded (n= 48)

♦  Not meeting inclusion criteria (n=48)

♦  Declined to participate (n=0)

♦  Other reasons (n=0)

Assessed for eligibility (n= 107)

**Educational Leaflet; S2**


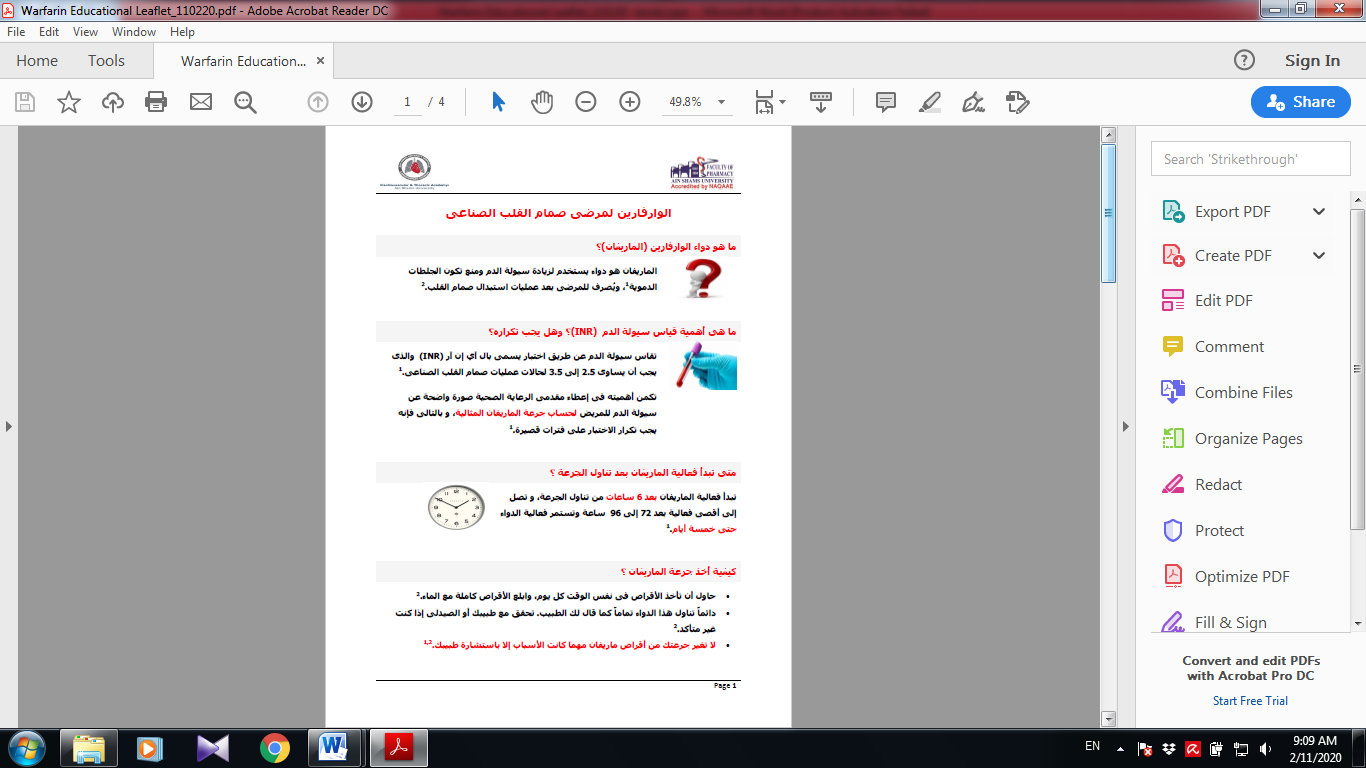


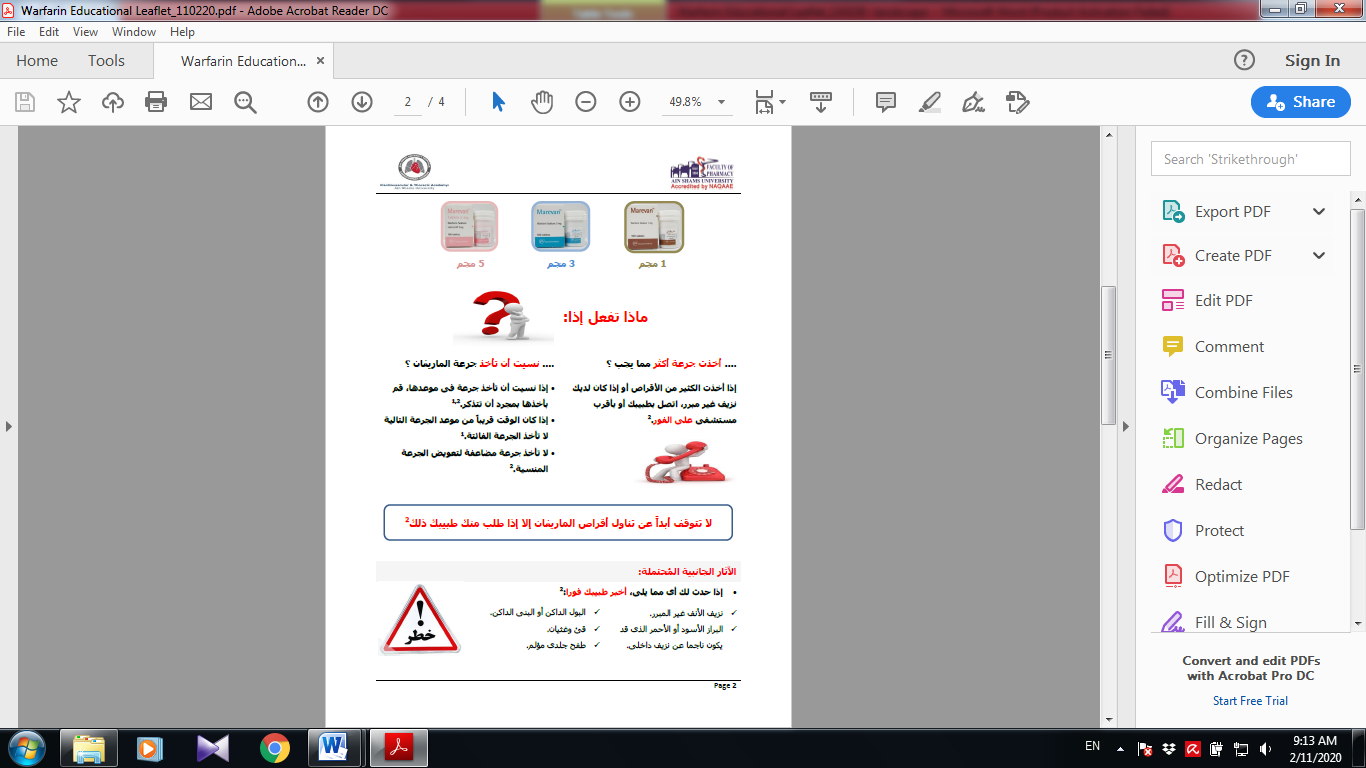


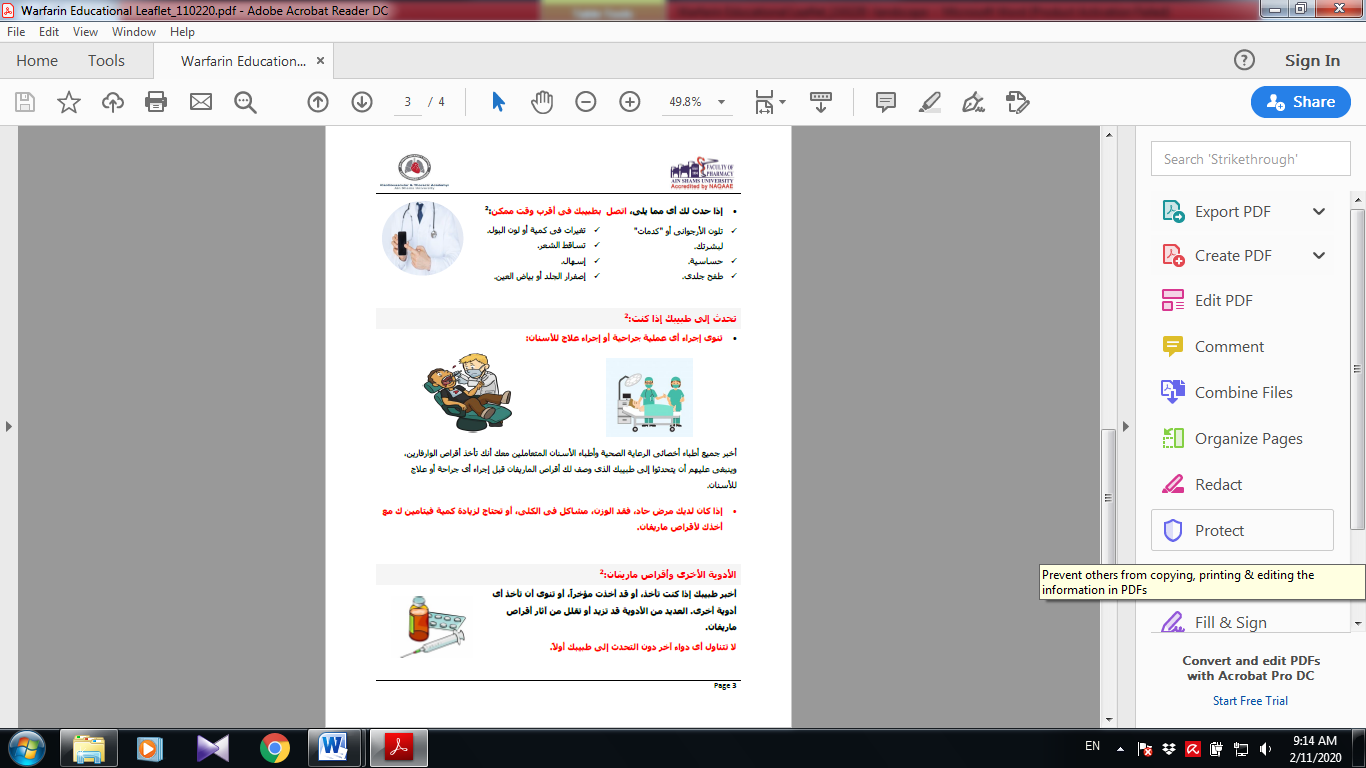


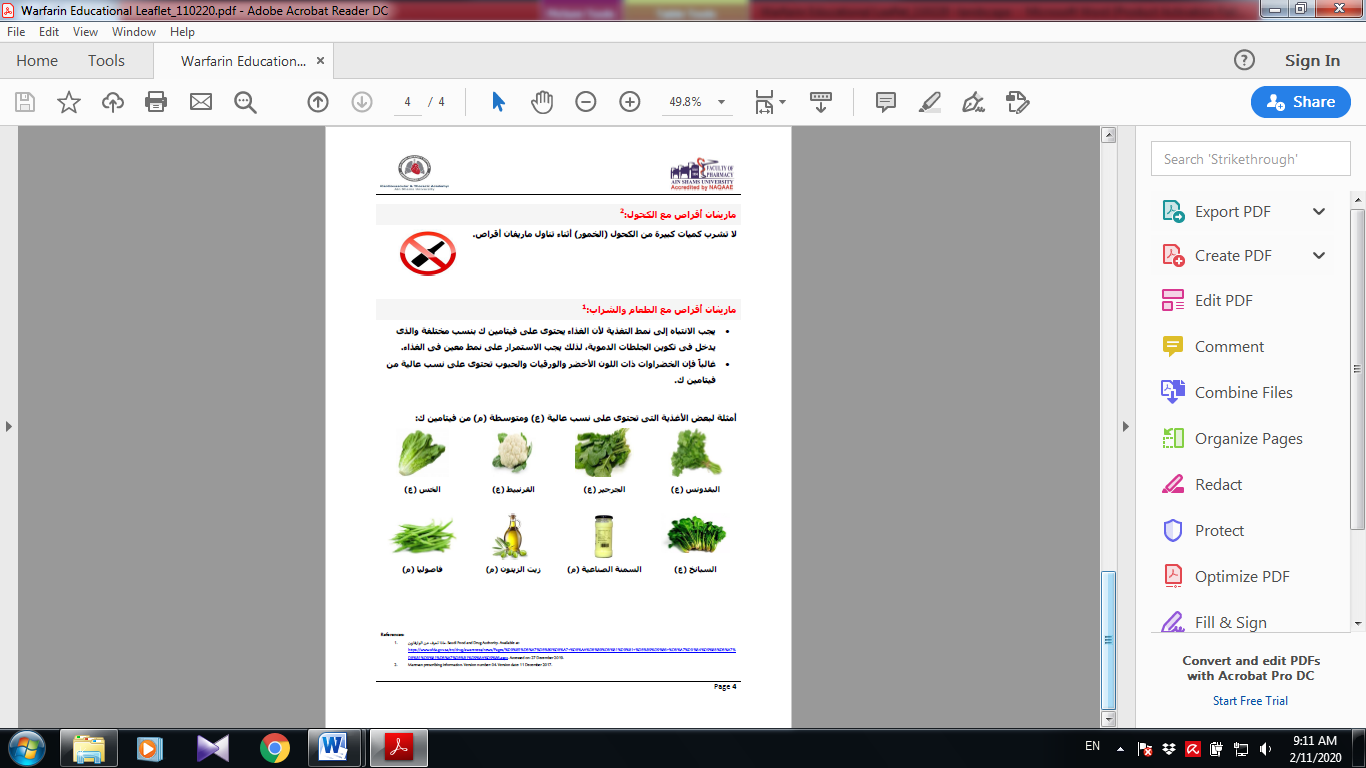


**Sample Size Calculation and Statistical Methods; S3**

The primary objective of this study was to compare the Time in Therapeutic Range (TTR) between patients in the intervention group who received standard medical care plus the clinical pharmacist‐provided services at the Warfarin Counseling Clinic, and patients in the Control group who received standard medical care only. Reference to Falamić et al., 2018, the median TTR was significantly higher in the group that received pharmacist‐provided services than the standard care group (93% vs. 31.2%, difference 61.8%). In the present study, we assumed a smaller difference in TTR (to decrease the effect size and increase the sample size) between the two groups of 30% (80% in the intervention group and 50% in the control group) with common standard deviations of TTR means in the two groups equal to 0.25, 80% power, and α-level of 0.05, a sample size of at least 32 patients (16 per group) with allocation ration 1:1 was required. We were expecting 10% drop out ratio, a total of 36 patients (18 per group) was requited. Sample size was calculated using Stata/ES 14.2 software for windows.


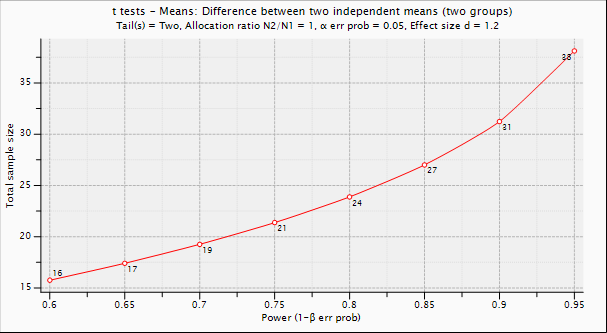


**Study parameters:** alpha = 0.0500, power = 0.9000, delta = -0.3000, m1 = 0.8000, m2=0.5000, sd = 0.2500

**Estimated sample sizes:** N = 32, N per group = 16

Statistical analysis of the study results was performed using IBM SPSS program, version 20. All graphs were done using Microsoft Excel 2010. Kolmogorov-Smirnov test was used for testing normality. Quantitative data were represented as mean ± standard deviation if they were normally distributed. Quantitative data which wasn’t normally distributed was represented as median (interquartile range IQR). On the other hand, describing qualitative data was represented as frequencies and percentages. Independent Sample T-test and Mann-Whitney test were used to compare means of quantitative variables with normal distribution and medians (IQR) of quantitative variables which were not normally distributed, respectively. For qualitative variables, Chi-square test was used to examine differences within the study participants. A multivariate logistic regression analysis was used to calculate the odds ratio (OR) with the 95% confidence interval (95% CI) of the association between standard care and poor anticoagulation control (i.e. TTR <65%). The following variables were used as covariates: age, BMI, number of comorbidities, heart failure, stroke, and diabetes. All *p*-values were two-sided, and the difference was considered statistically significant if *p*-value ≤0.05 (95% CI).

**Fig. S4**

**Median TTR in both Intervention and Control Groups**


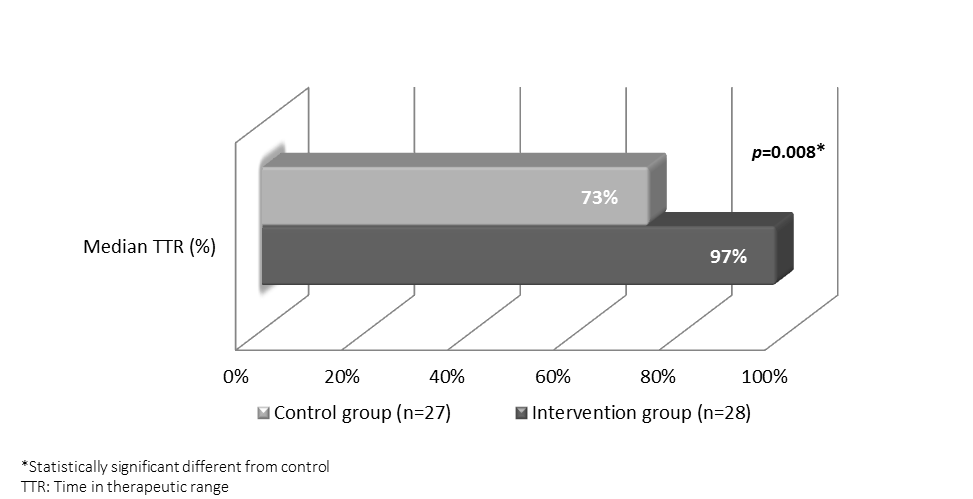


**Fig. S5**

**TTR Categories in both Intervention and Control Groups**

**Fig. S6**

**Forest plot displaying adjusted odds ratios resulting from the multivariable logistic regression analysis for poor anticoagulation control (dependent variable was TTR <65%)**
